# Influence of physico-chemical properties of hydroxypropyl methylcellulose on quetiapine fumarate release from sustained release matrix tablets

**DOI:** 10.1186/s13065-024-01311-2

**Published:** 2024-11-07

**Authors:** Takwa E. Ellakwa, Ahmad S. Abu-Khadra, Doha El-Sayed Ellakwa

**Affiliations:** 1 Physical Chemistry, Faculty of Pharmacy, Egyptian Russian University, Cairo, Egypt; 2 Basic Science Department, Faculty of Engineering, Sinai University, Al-Arish, Egypt; 3 Department of Biochemistry and Molecular Biology, Faculty of Pharmacy for Girls, Al-Azhar University, Cairo, Egypt; 4 Department of Biochemistry, Faculty of Pharmacy, Sinai University, Kantara Branch, Ismailia, Egypt

**Keywords:** Hydroxypropyl methylcellulose K15M, Quetiapine fumarate, pH, Hydrogel, Differential scanning calorimetry, Scanning electron microscope

## Abstract

Quetiapine fumarateis a typical antipsychotic with a short half-life of 6 h and is administered multiple times daily. In this study, a copolymer for controlled delivery of quetiapine fumarate will be developed. In order to prevent side effects and improve patient compliance, hydroxypropyl methylcellulose K15M (HPMC K15M) was included in the formulation of the quetiapine fumarate oral sustained-release tablets at a concentration of 10–30%. A series of analytical methods were used to determine the characteristics of the prepared hydrogels, including Fourier transform-infrared spectroscopy, Differential scanning calorimetry, X-ray diffraction, and Scanning electron microscope. At two different pH values (1.2 and 6.8), swelling and release studies were conducted. A variety of release kinetic models was used to study drug release mechanisms. A non-Fickian diffusion mechanism released hydrogels prepared from quetiapine fumarate. It was found that swelling was increased by increasing the amount of HPMC K15M. Compared to the other batches (10–20%), the produced tablets with 30% HPMC K15M content had a better release profile after 20 h of dissolution. Because of the effective matrix complex’s limited solubility in water, the drug diffuses through the gel layer at a steady rate rather than dissolving quickly.

## Introduction

**Fig. 1:**
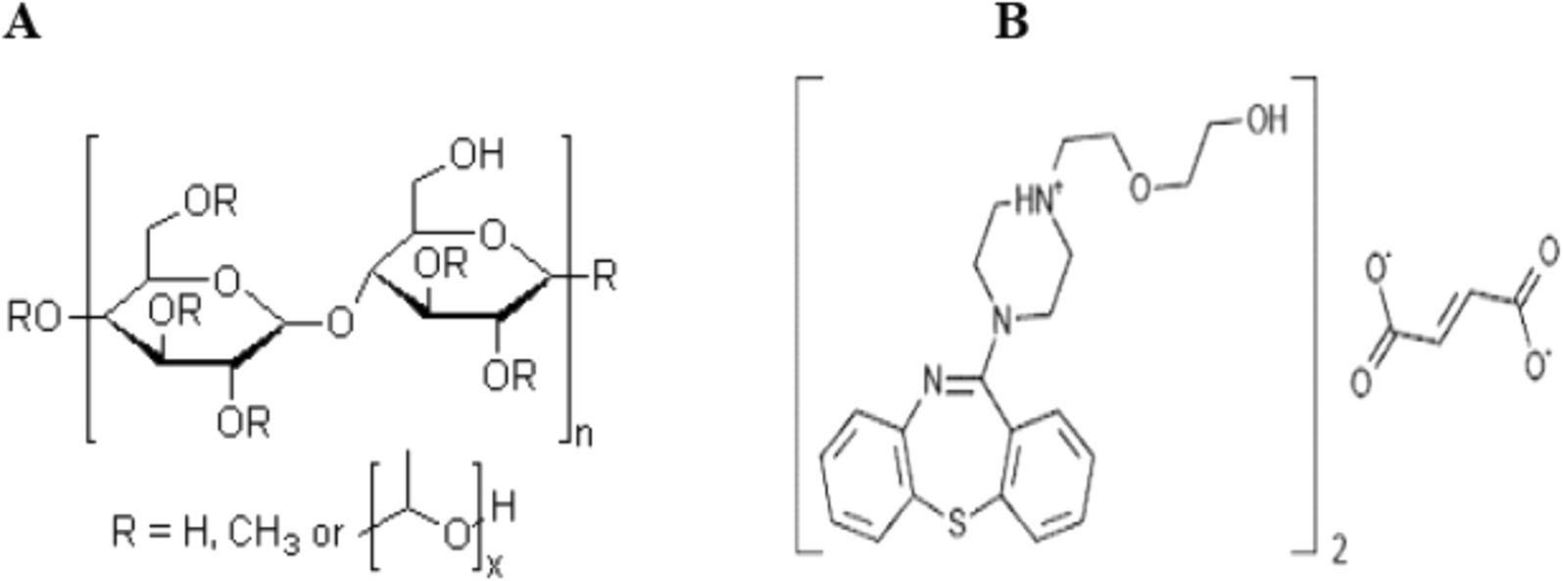
**A** Structure of hydroxypropyl methylcellulose. **B** Structure of quetiapine fumarate

Over the last 30 years, sustained release (SR) technology has quickly become a new interdisciplinary field that presents innovative methods for delivering bioactive agents into systemic circulation at a predetermined rate [7, 40]. Sustained-release formulations can achieve ideal therapeutic outcomes, long-lasting effectiveness, and reduced toxicity by maintaining a consistent for an extended period of time, and reproducible drug release rate [25, 29, 30]. Oral sustained-release hydrophilic matrix tablets are commonly used in gastrointestinal therapy (GI) [14, 18]. They allow for accurate control of drug release by the hydration of their polymer components. These tablets are cost-effective, offer flexibility in achieving the desired drug release profile, and promote patient compliance by providing a uniform therapeutic effect in the bloodstream over an extended period, which is impossible with traditional systems [5, 27]. Recently, researchers have been interested in using hydrophilic polymers for sustained drug release [38]. Natural polysaccharides are the preferred option among these polymers because they are non-toxic, biocompatible, biodegradable, and approved by regulatory agencies [1, 4]. Hypromellose, or hydroxypropyl methylcellulose (HPMC K15M), is an off-white or colourless powderthat does not have an odour or taste [6]. HPMC K15M has captured the attention of scientists and academics alike due to its exceptional biocompatibility and low toxicity, making it a promising candidate for various biomedical applications [1, 35, 36]. Different viscosity grades of hydroxypropyl methylcellulose, a thickening agent, are available, ranging from 4000 to 100,000 mPas [1, 2]. The sustained release patterns of HPMC matrices are attributed to two mechanisms, namely, the diffusion and erosion of the gel layer [4, 25]. The polymer viscosity affects the diffusion pathways [4, 6]. Hydroxypropyl methylcellulose, a high molecular weight polymer, the structure represent in (Fig. 1A), swells quickly and forms a thicker viscous layer than low molecular weight versions [3, 16]. The polymer chain relaxation influences the drug release rate in matrix products, leading to sustained release characteristics [3, 24]. Because HPMC K15M had the ability to form a gel, its disintegration was impeded in tablet form [32, 34]. Quetiapine fumarate is the most prevalent antipsychotic drug that’s used to treat bipolar disorder and schizophrenia. Quetiapine fumarate is a dibenzothiazepine derivitve, the structure represent in Fig. 1B. The present study aims to develop a sustained-release formulation of quetiapine fumarate, a drug with higher solubility under acidic conditions due to its dibenzo thiazepine structure with two basic nitrogen atoms [7, 37]. Quetiapine fumarate has a mean elimination half-life of approximately 6 h, requiring administration twice or thrice daily to maintain therapeutic plasma levels [29, 40]. A pH-dependent polymer was used to extend the drug’s release due to the pH changes that occur during the transition from the stomach to the colon [17, 39]. Lin et al., suggest that a once-daily controlled-release formulation of quetiapine fumarate could improve patient compliance and clinical efficacy [12, 26, 32]. This study’s novelty is to use the lowest concentration of hydroxypropyl methylcellulose K15M as a polymer with quetiapine fumarate to decrease the solubility of a drug to prepare a sustained-release formulation.

## Materials and methods

### Materials

All the materials used in the study were pharmaceutical-grade and were purchased from different suppliers. The active drug, Quetiapine fumarate, was obtained from Hetero Drugs Limited (India) and had a purity of 98.0%–102.0% as required by the USA Pharmacopeial Forum. Hydroxypropyl methylcellulose (HPMC K15M) was obtained from Sigma Aldrich (Darmstadt, Germany), while Polyvinyl Pyrrolidone K30 (PVP k30), Microcrystalline cellulose (trade name is Avicel® PH-102), Magnesium Stearate, and Talc were all obtained from International Specialty Products (Germany).

### Methods

#### Formulation of quetiapine fumarate SR tablets by Wet Granulation

A mixture of Quetiapine fumarate, Avicel PH 102, and HPMC K15M (trade name METHOCEL™) was granulated in double cone blender macine (India) using polyvinyl pyrrolidone K 30 in ethanol 70% as the granulating fluid. The final wet granulate was dried at 55–60 °C in an oven and checked for less than 2% Loss on Drying (LOD) before being sieved through a No. 12 Mesh Screen. The dried granules were then mixed with talc, magnesium stearate, and Colloidal silicon dioxide 200 in a mini drum blender for 2 min. Using an Oval biconvex punch 21 × 9.3 mm tooling, the mixture was compressed with the blending using atablet press machine (Korsch EK-0, Germany), targeting a weight of 970 mg. Then, compressed tablets were coated. The preparation of the coating solution is as follows (dissolve polyethylene glycol 6000 in isopropyl alcohol, then mix each of Hydoxypropyl methylcellulose 2910, talc and titanium. The quantity and ingredients used in all formulations are shown in Table 1.

**Table 1:** Composition (mg) of quetiapine fumarate tablet formulations

Name of ingredient	Formulation A (mg)	Formulation B (mg)	Formulation C (mg)
Intergranular			
Quetiapine fumarate (equivalent to quetiapine base 300 mg)	345.4	345.4	345.4
Hydroxypropyl methylcellulose K15M	90.0	60	30
Microcrystalline cellulose PH 102	406	436	466
PVP k 30	50.0	50.0	50.0
Extra granular			
Colloidal silicon dioxide 200	19.0	19.0	19.0
Talc	19.6	19.6	19.6
Magnesium stearate	20	20	20
The total weight (mg)	950.00	950.00	950.00
Film coat			
Hydroxypropyl methylcellulose 2910	19.10	19.10	19.10
Talc	0.3	0.3	0.3
Titanium dioxide C.I.N: 77891	0.3	0.3	0.3
Polyethylene glycol 6000	0.3	0.3	0.3
The total weight (mg)	970.00	970.00	970.00

#### Drug-excipient compatibility study

##### Fourier transform infrared spectrometer (FTIR)

The Schmooze (FT-IR 8400S spectrometer, UK)was used to obtain FTIR spectra, which were taken at a resolution of 2 cm^−1^ with potassium bromide as a reference. Before scanning, the samples, including Quetiapine fumarate, Hydroxypropyl methylcellulose, and a physical mixture of drug and polymer (1: 0.3), were ground and mixed thoroughly with potassium bromide. Then, discs were created from the resulting powders using a hydraulic press.

##### Differential scanning calorimetry (DSC)

In a differential scanning calorimeter DSC 2920 (TA Instruments, USA) with a thermal analyzer, DSC measurements of quetiapine fumarate, hydroxypropyl methylcellulose, and a physical mixture of quetiapine fumarate with hydroxypropyl methylcellulose (1:0.3) were taken. A nitrogen flow rate of 50 mL min^−1^ was used to heat samples weighing around 3 mg from a temperature of 25 °C in sealed aluminium pans.

##### X-Ray diffraction of powder

The XD-6000 (Shimadzu, Japan) powder x-ray diffractometer was used to record the powder x-ray diffraction patterns. The samples were subjected to radiation analysis at a 40 kV voltage and a 40 mA current, with a heating rate of 2 °C min^−1^. This technique has been employed in every research studies referring to the characterization of solid dispersion because it is essential for determining the level of crystallinity of solids.

##### Scanning electron microscope (SEM)

To examine the morphology of quetiapine fumarate with polymer, a Scanning electron microscope was employed (JSM-6360, Jeol Ltd., Japan). A monolayer dry microsphere was fixed on an aluminium slab using double-side carbon tape, and the sample was coated with a 10 nm thick gold film using a sputter coater. The coated sample was then examined using an electron acceleration voltage of 10 kV [14].

#### Evaluation of tablets of quetiapine

##### Pre-compression parameters

Flowability testing: the produced powders were examined for flowability by measuring their angle of repose, compressibility index, and Hausner’s ratio. The average standard deviation for experiments is three.

Angle of repose measurement: the fixed height cone method was used, and the resulting cone’s (d) diameter was calculated using the formula below:

$${\text{Tan}} \;\uptheta = 2{\text{h}}/{\text{d}}.$$

Determination of the initial and tapped bulk densities: with no outside force used, a fixed weight of the drug powder was poured into a 25 mL graduated cylinder, allowed to settle, then measured as V_I_ (initial bulk volume). The cylindrical graduate was then tapped on a plane surface at a one-inch distance till a constant volume was obtained. The tapped volume of the powder was then recorded as (V_T_). The initial and tapped bulk densities were then calculated using the following equation[2, 8].

$${\text{Density}}\;{\text{of}}\;{\text{initial}}\;{\text{Bulk}}\;\uprho _{{\text{I}}} = {\text{M}}/{\text{V}}_{{\text{I}}} ,$$

$${\text{The}}\;{\text{Density}}\;{\text{of}}\;{\text{tapped}}\;{\text{bulk}}\;\uprho _{{\text{t}}} = {\text{M}}/{\text{V}}_{{\text{T}}} ,$$

where (M) is the mass of the powder.

Hausner’s ratio: flow properties of granules are determined by the ratio of tapped density to bulk density.

The percentage Hausner’s Ratiowere then determined from the following equation

$${\text{Hausner}} {\text{s}}\;{\text{Ratio}} = {\text{Tapped}}\;{\text{density}}/{\text{Bulk}}\;{\text{density}},$$

$${Hausners Ratio} =\uprho _{{\text{t}}} /\uprho _{{\text{b}}} .$$

ρ_t_ = tapped density; ρ_b_ = bulk denisty.

Compressibility index (Carr’s Index): one significant metric that may be derived from the bulk and tapped densities is the compressibility index. Theoretically, a material becomes more flowable the less compressible it is. The percentage compressibility (Carr’s index) were then determined from the following equation [2, 8, 9]

$${\text{Carr}} {\text{s}}\;{\text{index}} = 100\;(\uprho _{{\text{t}}} -\uprho _{{\text{b}}} /\uprho _{{\text{T}}} ).$$

Weight variation tests: twenty tablets selected at random were weighed, and the average weight was calculated.

Hardness: a tablet’s hardness reveals if it can tolerate mechanical shocks while being handled. To determine the hardness of the tablets, (a Dr. Scheuniger hardness tester, USA) was used, and the results were expressed in Newtons (N). The hardness of ten randomLy selected tablets was determined.

Thickness: tablets were selected and measured for thickness and diameter using the digital vernier calipers.

Friability test: the tablets’ friability was assessed using the friability tester (Erweka TA 100, USA). It is expressed in percentage terms. A total of ten tablets were weighted (W_initial_) and then transferred to the friabilitor. The friabilitor was operated at 25 rpm for 4 min. The tablets were weighted again (W_final_). By using this equation, we were able to calculate the percentage of friability.

$${\text{F}} = \left( {{\text{W}}_{{{\text{initial}}}} {-}{\text{W}}_{{{\text{final}}}} } \right){\text{/W}}_{{{\text{initial}}}} \times 100.$$

Percentage of Friability of tablets less than 1.0% is considered acceptable.

Swelling index: as polymeric matrix tablets come into contact with water, a gel layer forms around the tablet core. This gel layer controls how the drug is released. The gel barrier is created by water penetration. Hence, the dynamics of swelling are significant. The swelling index was studied for three formulations up to 20 h at different pH (1.2, 6.8) and different concentrations of polymer of HPMC k15M. Tablets were initially weighed and carefully placed on Petri dishes. The temperature is maintained at about 37 °C [2, 22]. Tablets were cleaned with tissue paper to remove extra solution and weighed after regular intervals of time. The tablets were weighed and then added back to the solution. Variation in weight is indicative of the quantity of water taken up by the polymer. The Swelling index for each table is determined using the formula.

$${\text{Swelling}}\;\% = \left( {{\text{M}}_{{\text{t}}} {-}{\text{M}}_{0} } \right)/{\text{M}}_{0} \times 100,$$

M_t_ = tablet weight at time t; M_0_ = table weight at time t = 0.

#### Drug content assay test

Twenty tablets were weighed and powdered. Quetiapine powder equivalent to a single dose was weighed, and its drug content was analyzed using a Benchtop UV/VisibleSpectrophotometer (Jenway 635 001; 230 VAC). In accordance with ICH guidelines, the UV method has been validated (Q2 R1).

#### In vitro drug release study

Tablet dissolution was assessed in using a standard apparatus dissolution test apparatus (Agilant-Evolution 708-Dissolution Sampler—USA) employing the baskets method. The dissolution test was performed according to USP 40 monograph (Table 2) using two different mediumsin which medium 1:900 mL 0.1 N HCl of pH 1.2 for the first hour maintained at 37 ± 0.5 °C. Medium 2: Dissolve 17.9 dibasic sodium phosphate dodecahydrate in 400 mL of water. Add 460 mL of 1.0 N sodium hydroxide and dilute with water to 1.0 L (1000 mL), pH 6.8. The speed of stirring was 200 rpm (Stirrer LH, Velp Scientifica). Samples were collected at each time point (1, 6, 12, and 20 h). 1.0 mL of sample was collected and filtered through 10 μm dissolution filters [2]. Following the collection of each sample, a fresh dissolution medium was added. The % release of the drug was analyzed at 248 nm using UV/Vis spectrophotometer [2, 13, 15].

**Table 2:** Dissolution profile for quetiapinefumarate tablet according USP 40 monograph

Time point	Time (h)	Amount dissolved (%)
1	1	NMT 20
2	6	47–69
3	12	65–95
4	20	NLT 85

#### Stability study

The stability investigation was carried out according to the International Conference on Harmonization ICH Q1A guideline [21]. Formulation A tablets were chosen for stability studies. Stability testing provides information on how the quality of a drug product changes over time under the effect of different environmental elements including temperature, humidity, and light, which is useful in defining optimum storage conditions and shelf life. The tablets were stored in airtight polyethylene bottles for 6 months and subjected to an accelerated stability study in a stability chamber (Thermo Scientific™ Vertical Light Chambe, USA at 40 °C and 75 percent relative humidity. The tablets were taken out at these times so that properties such as color, water content, in vitro drug dissolution, and percent drug content could be analyzed [2, 9].

#### Kinetics of drug release

Several release profiles were tested, including zero order, first order, Higuchi and Korsmeyer Peppas equations (Eqs. 1–4) to evaluate the in vitro drug release profiles of Formulation A. The mathematical method models were fitted to each dissolution data using open software [10, 19, 23]. The release studies’ experimental results were fitted using.

1. Zero-order model
  1 $${\text{C}}_{{\text{t}}} - {\text{C}}_{0} = {\text{K}}_{0} {\text{t}}{.}$$
  The drug released at time t is Ct, the initial amount of drug in solution at time t = 0 is represented by C_0_ and the zero-order rate constant is K_0_.
2. First-order model
  2 $$\log \;{\text{C}} = \log \;{\text{C}}_{0} - {\text{K}}_{1} {\text{t}}/2.303.$$
  C is the drug’s remaining amount at time t, C_0_ is the drug’s starting concentration, and K_1_ is the first order rate constant.
3. Higuchi’s model
  3 $$Q = A\sqrt {D\left( {2C_{0} - C_{S} } \right)} C_{S} t.$$
  Q is the total amount of drug released in time t per unit area. C_0_ is the initial drug concentration, D is the drug’s diffusion coefficient in the matrix and C_S_ is the drug’s solubility in the matrix.
4. Korsmeyer Peppa’s model
  4 $$\log \left( {{\text{Mt}}/{\text{M}}\infty } \right) = \log \;{\text{K}}_{{\text{p}}} + {\text{n}}\;\log \;{\text{t}}{.}$$
  Mt is the total amount of drug released in time t, M∞ is the total amount of drug released after time ∞ and K_p_ is the Korsmeyer release rate constant.

## Results and discussion

### Formulation of quetiapine fumarate SR tablets

The present study used wet granulation to prepare quetiapine fumarate SR matrix tablets according to (Table 1). In total, three formulations were developed. The batch size for each formulation is three kilogram. It was confirmed that the prepared SR matrix tablets of quetiapine fumarate met standard Pharmacopeial requirements.

### Drug-excipient compatibility study

#### Infrared spectral analysis

**Fig. 2:**
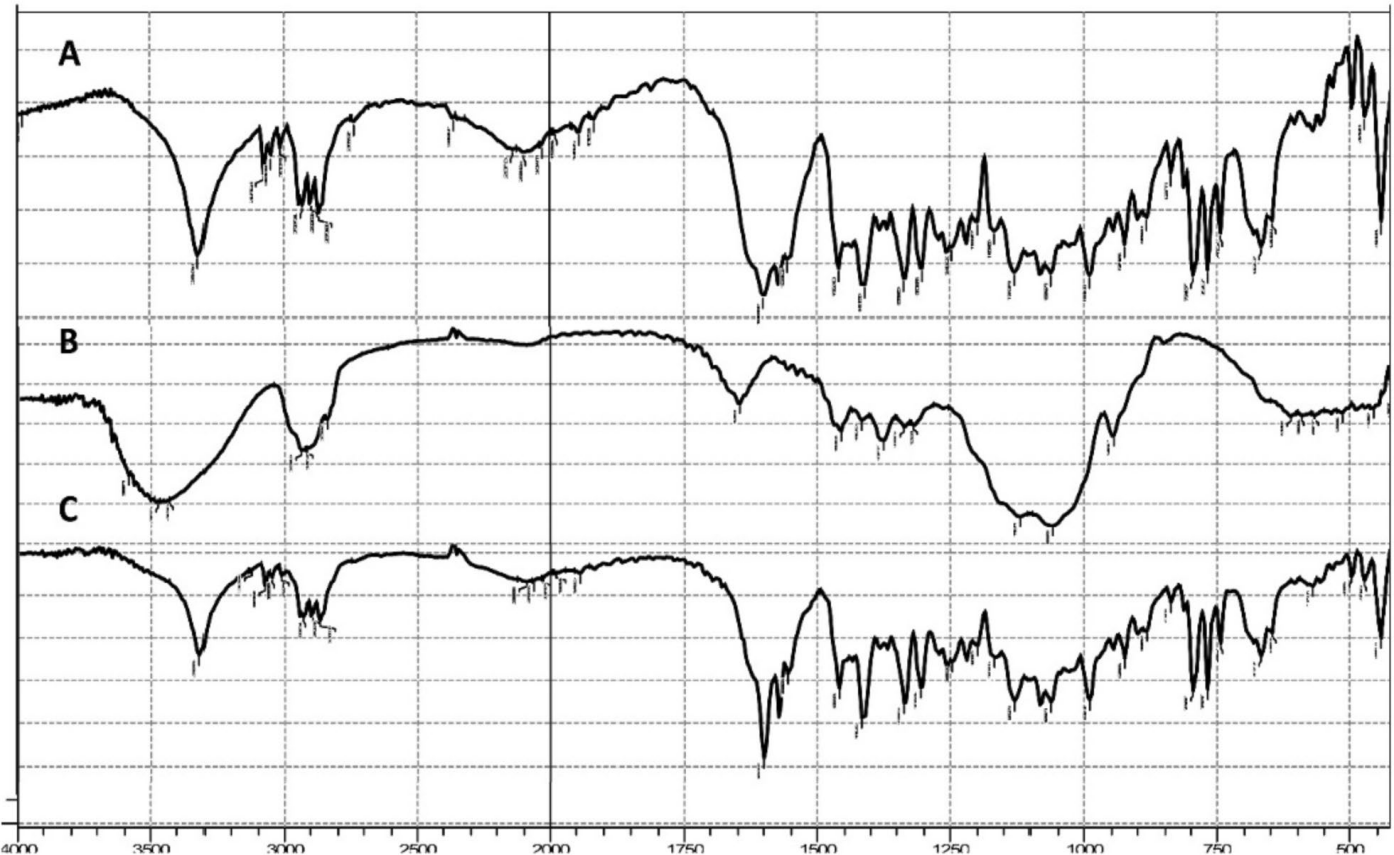
IR spectra of** A** quetiapine fumarate,** B** polymer and** C** physical mixture

The IR spectrum of quetiapine fumarate (Fig. 2A) shows a characteristic a peak at 3317 cm^−1^ due to O–H stretching and a peak at 2870 cm^−1^ due to C–H aliphatic stretching, a peak at 3074 cm^−1^ due to C–H aromatic, peak at 1600 cm^−1^ due to C=N, peak 1580 cm^−1^ due to C=C [28, 33]. The IR spectrum of hydroxypropyl methylcellulose (Fig. 2B) shows a characteristic peak at 3475 cm^−1^ due to OH and a peak at 2935 cm^−1^ due to C–H aliphatic stretching [2, 34]. The IR spectra of the quetiapine fumarate and hydroxypropyl methylcellulose physical mixture (Fig. 2C) reveals no new peaks appearing or old peaks disappearing, proving there is no drug-polymer interaction [23, 29].

#### X-Ray powder diffraction

**Fig. 3:**
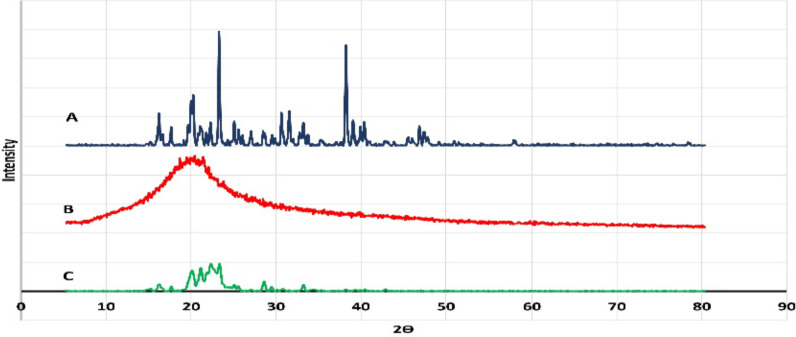
X-Ray of** A** quetiapine fumarate,** B** polymer and** C** physical mixture

The X-ray diffraction pattern of quetiapine fumarate (Fig. 3A) indicates that quetiapine fumarate is a crystalline material with characteristic diffraction peaks [31, 38]. The X-ray diffraction pattern of hydroxypropyl methylcellulose (Fig. 3B) indicates that hydroxypropyl methylcellulose is a non-crystalline material [1, 13]. The X-ray diffraction pattern of the physical mixture of quetiapine fumarate with polymer (Fig. 3C) indicates that some height peaks were decreased. The overall diffraction pattern revealed no change in the polymorphic properties of quetiapine fumarate [22, 28].

#### Differential scanning calorimetry (DSC)

**Fig. 4:**
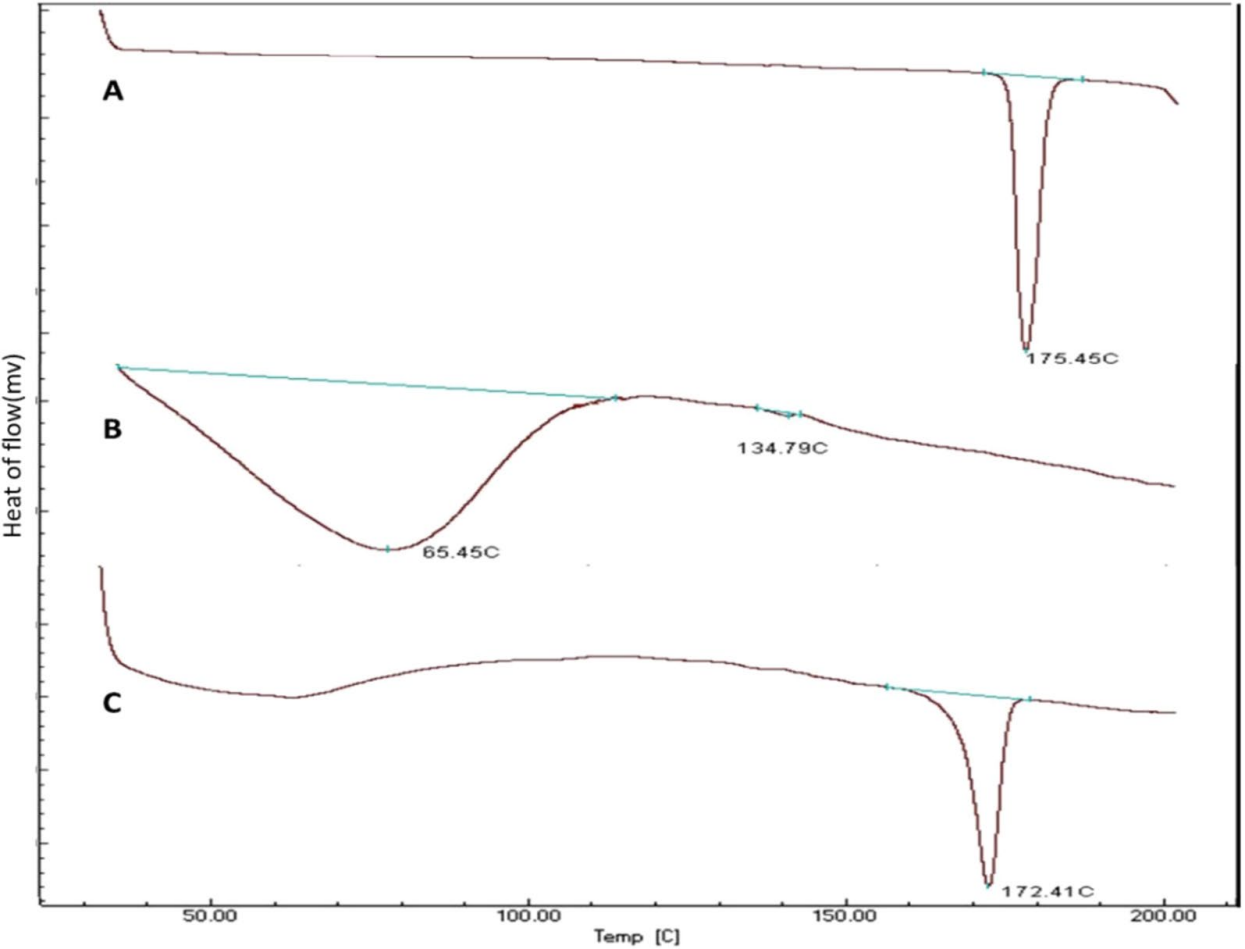
DSC thermogram of** A** quetiapine fumarate,** B** polymer and** C** physical mixture

DSC study was performed on the pure quetiapine fumarate, HPMC K15M polymer, and Physical mixture of quetiapine fumarate with polymer (Fig. 4A–C respectively). The thermogram of pure quetiapine fumarate gave a melting endotherm at 175.45 °C [31]. The thermogram of HPMC K15M give a melting endotherm at 65.45 °C [1]. The thermogram of the Physical mixture of the drug with polymer gave a melting endotherm at 172.41 °C. So from the DSC curve, it has been observed that there is no change in the endothermic peak of the drug, and hence the drug and polymers are well compatible [22, 23].

#### Scanning electron microscope (SEM)

**Fig. 5:**
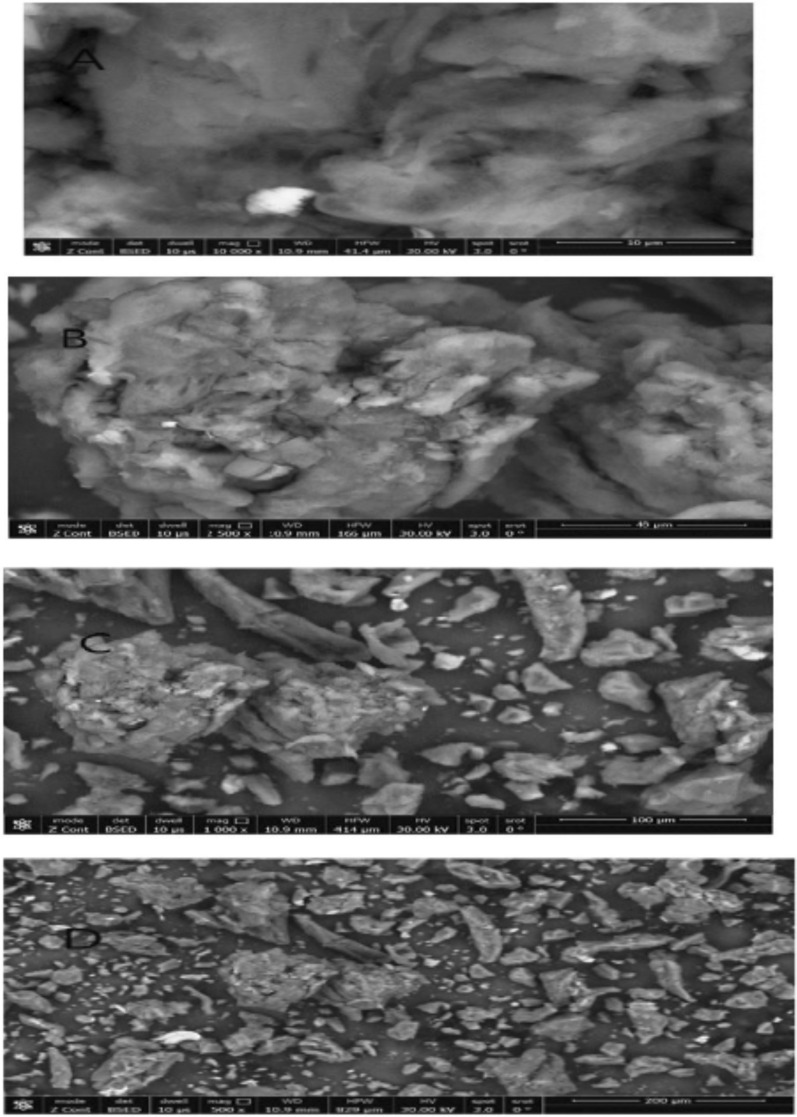
SEM of quetiapine fumarate formulation A (**A**–**D**)

The surface morphology of quetiapine fumarate with polymer (formulation A) was examined using scanning electron microscopy. Figure 5 suggests that the hydrogel disc had a super-porous surface and a rough surface. According to the SEM image, portray A and B, the polymer matrix contains numerous capillary channels connected by interconnected pores. Water molecules can slowly permeate into the polymer matrix through the capillary channels, dissolve quetiapine fumarate, and then release it over time in a sustained manner. As shown in portray C and D, the rough surface has pores and crevices, entrapping drug particles in the pores and crevices. This cause the release of drug particles to retarded [14, 27].

### Physicochemical evaluation of prepared tablets formulation A

The values of the physical parameters of prepared tablets were found to be within acceptable limits, and the pre-compression parameters (Table 3) were found to be satisfactory. Physical parameters of the quetiapine fumarate matrix tablets, including thickness, diameter, weight variation, hardness, friability, and drug content, were assessed (Table 4).

**Table 3:** Precompessional parameters of formulation A

Parameters	
The angle of repose	29.1 ± 0.05
Bulk density (g/cm^3^)	0.30 ± 0.02
Tapped density (g/cm^3^)	0.33 ± 0.01
Carr’s index	9.09 ± 0.10
Hausner’s ratio	1.1 ± 0.01

**Table 4:** Physical parameters of the prepared tablets

Parameters	
Weigt variation (mg)	970 ± 01
Hardness (kg/cm^2^)	7.1 ± 0.3
Thickness (mm)	9.3 ± 0.4
Friability (%)	0.52 ± 0.02

### Swelling index of prepared tablets

**Fig. 6:**
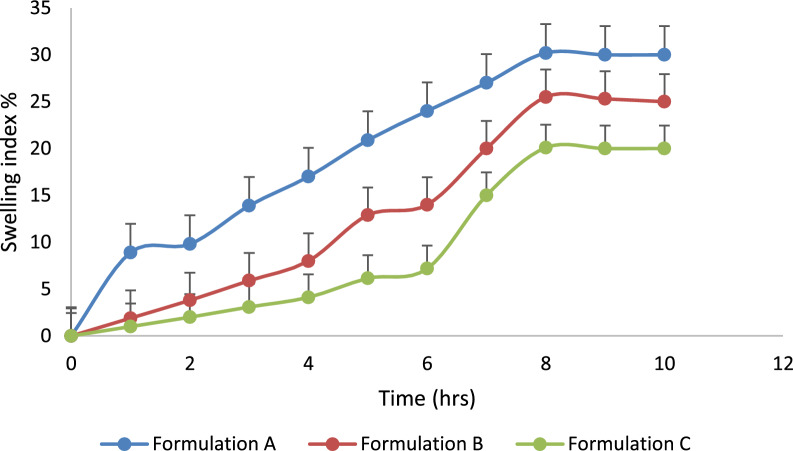
Swelling index of prepared tablets at pH 1.2

**Fig. 7:**
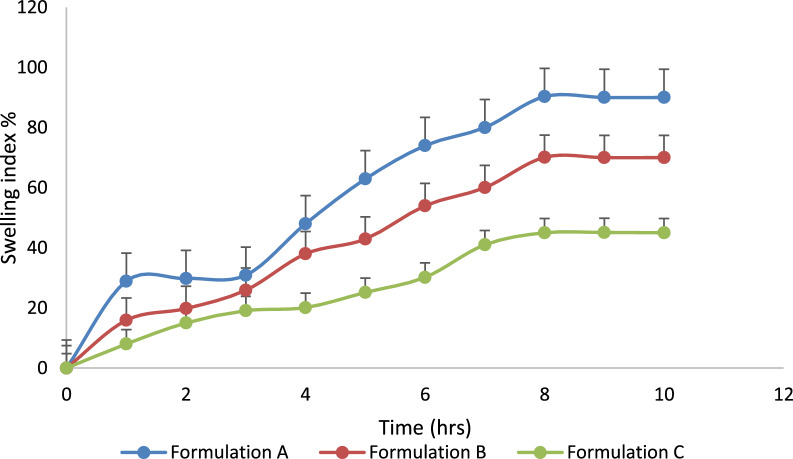
Swelling index of prepared tablets at pH 6.8

The drug is released from the polymer matrix when the medium’s pH interacts with the polymers and causes the swelling behavior [2, 20]. The study of swelling behavior showed the rate at which matrix tablets absorb the water from the dissolution media and swell up. At pH 1.2, the water uptake ratio of all three formulations was low and independent of time. The swelling index of all formulations at pH 6.8 increased proportionally to time increase and reached its peak at 8 h. No notable morphological changes were observed in the tablets during our investigation. The swelling index (formulation A was higher as compared to the swelling index of formulation B and formulation C. From data of Figs. 6, 7, the value of swelling % increased with increased polymer and alkaline medium concentration. The swelling index showed a maximum at 8 h at pH 6.8. This behavior of swelling is the best of prepared sustained release formulation.

### Drug content assay test

Each film-coated sustained-release tablet contains 97.24 mg (97.24 ± 1.80) of quetiapine fumarate per 100 mg of tablet.

### In vitro drug release study

**Fig. 8:**
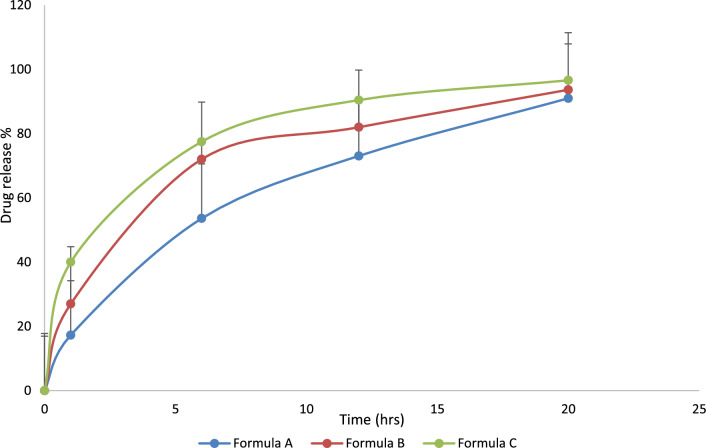
In-vitro drug release of formulation A, formulation B and formulation C

The dissolution study for all formulations was carried out in 0.1 N HCl pH 1.2 for 1 h. As well as up to 20 h in phosphate buffer pH 6.8. The dissolution profile of the three prepared formulation (A, B, C) tablets are shown in Fig. 8. In this study, we calculated dissolution rates by calculating the percent of the dose dissolved within a sampling interval. The percentages of drug dissolved from Quetiapine fumarate (Formula A) containing 17.28%, 53.6%, 73.06%, and 91.0% after 1, 6, 12, and 20 h respectively.

After 1, 6, 12, and 20 h, the percentages of drug that had dissolved from quetiapine fumarate (Formula B) were 27%, 72.05%, 62.43%, 82.00%, and 93.66% respectively.

In formula C, the percentage of quetiapine fumarate that dissolved after 1 h, 6 h, 12 h, and 20 h was 40%, 77.5%, 90.45%, and 96.6% respectively. Faster drug release was observed from formulated patches containing low amounts of the hydrophilic polymer. Release studies revealed that polymer concentrations were higher in formula A**.** Therefore, formula A is likely to retard quetiapine fumarate dissolution more than formula B and formula C (Fig. 8). So quetiapine fumarate tablets contain the highest amount of a polymer, prolonging the release period by 12 to 20 h (Table 5). Since HPMC k15M is frequently utilised in hydrophilic matrix delivery systems, it creates a viscous gel when it comes into contact with aqueous solutions, which may be useful for the controlled delivery of medications that are very soluble in water. Because of the increase in HPMC k15M viscosity, the drug’s release was extended. The efficient and quick release of the medication from the hydrophilic matrices may have resulted from the efficient and quick dissolving of the water-soluble pharmaceuticals within the spheres, which are now spreading outward and creating pores that allow solvent molecules to enter [22, 23].

**Table 5:** Result of optimized formulations A

Parameters	Results	
1. Appearance	Oval biconvex film coated tablets	
2. Color	White	
3. Average weight (mg)	970.0 mg ± 5%	
4. Thickness (mm)	9.3 ± 0.4	
5. Hardness (kg/cm^2^)	7.1 ± 0.30	
6. Friability (%)	0.52 ± 0.02	
7. Drug content (%) Range (90–110% of the stated amount)	97.24 ± 1.80	

% Drug release		
Time (h)	Range	Result (%)
1	NMT 20%	17.28
6	47–69%	53.59
12	65–95%	72.93
20	NLT 85%	91.02

### Kinetic evaluation of drug release data

**Fig. 9:**
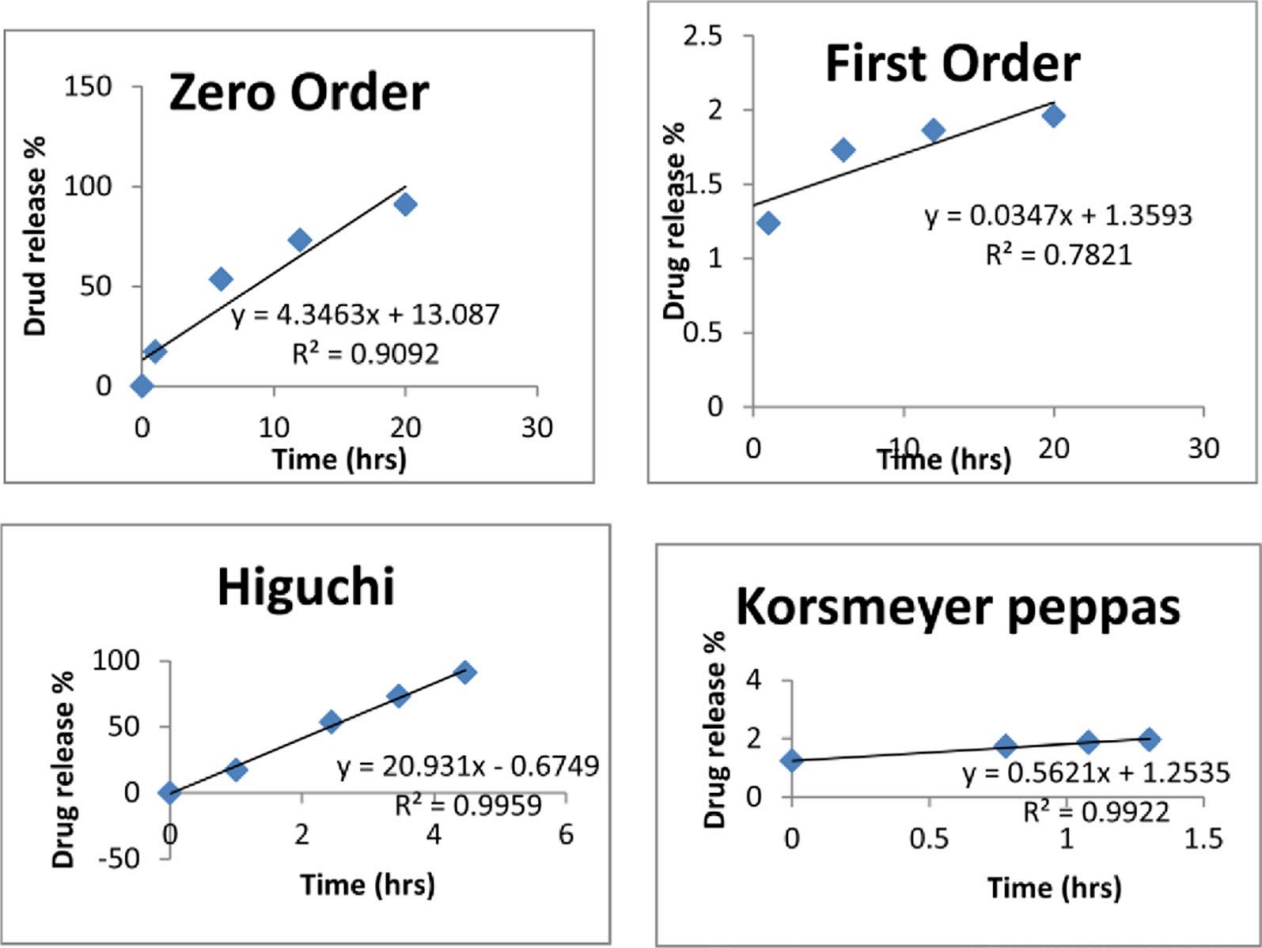
Drug-release kinetics profile of formulation A

The in vitro drug release profile (Fig. 9) was developed to investigate the hydrogel’s ability to release drugs. Kinetic modeling was fitted to Zero order, first order, Higuchi, and Korsmeyer Peppas to understand the mechanism of in vitro drug release (Formulation A). From the values of correlation factors (R^2^), (Table 6), the Higuchi model supported release data. This express non-Fickian diffustion mechanism [2, 11, 24].

**Table 6:** Drug release kinetic study of formulation A

Formulation A	Kinetics models			
	Zero order	First order	Higuchi	Korsmeyer Peppas
Y equation	y = 4.3463x + 13.087	y = 0.034x + 1.3593	y = 20.931 × 0.6749	y = 0.5621x + 1.2535
R^2^	0.9092	0.7821	0.9959	0.9922

## Stability tests

Six-month stability studies were conducted on formulation A at75% relative humidity and 40 °C (accelerated stability study). At the end of the 6 months, no significant changes had been observed in the physical appearance, and a hardness test was performed. A table with the results is shown below in Table 7. The in-vitro dissolution profiles of formulation A at 40 °C, and RH 75 percent ± 5 percent indicated 90.90 percent release after 1 month. A 90.50 percent release after 3 months. In 6 months, there will be an 89.46 percent release. Thus, the stability analysis revealed no improvement in physical appearance, stiffness, or drug content. In this study, formulation A was found to be stable at temperatures of 40 °C for 6 months.

**Table 7:** Results of accelerated stability studies for 6 months (temp. 40 °C ± 2 °C and RH 75% ± 5%)

Physical parameter	Initial	1 month	3 month	6 month
Appearance	White colored, oval tablet	White colored, oval tablet	White colored, oval tablet	White colored, oval tablet
Average weight (mg)	970 ± 3.30	975 ± 2.30	978 ± 2.10	980 ± 2.40
Friability (%)	0.52 ± 0.02	0.55 ± 0.01	0.59 ± 0.06	0.62 ± 0.11
Hardness (kg/cm^2^)	7.1 ± 0.30	7.3 ± 0.50	7.5 ± 0.70	7.9 ± 0.95
Thickness (mm)	9.3 ± 1.80	9.3 ± 1.80	9.3 ± 1.90	9.3 ± 2.30
Drug content (%)	97.24 ± 1.80	97.00 ± 2.30	96.98 ± 2.90	96.85 ± 2.90
Drug release after 20 h (%)	91.00 ± 0.764	90.9 ± 2.58	90.5 ± 3.10	89.46 ± 1.10

Values represent mean ± SD (standard deviation)

## Conclusions

This study was conducted to formulate and evaluate quetiapine fumarate SR matrix tablets for treating schizophrenia using a polymer HPMC K15M. Quetiapine fumarate was controlled and delivered with hydrogels whose release and swelling were pH dependent. Swelling and release are influenced by the amount of HPMC K15M present. It has been observed that as the concentration of HPMC K15M increases, the drug release decreases. Possibly, HPMCK15M has a slower erosion rate, which helped to keep the hydrated gel intact for 20 h, allowing the drug to be released. The results of the in vitro release data indicated that the concentrations of polymer had an impact on the drug release from the SR tablets. HPMC K15M demonstrated improved drug release at a lower concentration. According to FTIR, DSC, and XRD, a hydrogel was formed, which maintained its thermal stability better than individual ingredients. Additionally, gel fractions were directly proportional to HPMCK15M amounts. The HPMCK15M hydrogel had excellent mechanical strength, as well as the ability to withstand biological stress. It was determined from the findings that hydrogels can be created through a pH-dependent process and can intelligently react to their surroundings. These hydrogels can effectively administer quetiapine fumarate for Alzheimer-related dementia treatment, resulting in reduced dosing frequency and improved patient adherence. The accelerated testing for 6 months demonstrated the excellent storage stability of sustained release of quetiapine fumarate tablet.

The above results and discussion suggest that the formulation of SR matrix tablets shows a slow, sustained and complete release of quetiapine fumarate over a 20-h period.

## Data Availability

All data generated or analyzed during this study are included in this published article and its supplementary information files. Raw data can be shared via correspondence upon reasonable request.

## Abbreviations

HPMC K15M: Hydroxypropyl methylcellulose K15M
SR: Sustained release
LOD: Loss on drying
FTIR: Fourier transform infrared spectrometer
DSC: Differential scanning calorimetry
SEM: Scanning electron microscope
NMT: Not more than
NLT: Not less than
(ICH) Q1A: International Council for Harmonisation
RH %: Relative humidity
